# Older Lineages of Oribatid Mites in Mountain Ranges Have Broader Geographic Ranges and Exhibit More Generalistic Traits

**DOI:** 10.1002/ece3.71046

**Published:** 2025-02-28

**Authors:** Xue Pan, Bastian Heimburger, Ting‐Wen Chen, Jing‐Zhong Lu, Peter Hans Cordes, Zhijing Xie, Xin Sun, Dong Liu, Donghui Wu, Stefan Scheu, Ina Schaefer, Mark Maraun

**Affiliations:** ^1^ J.F. Blumenbach Institute of Zoology and Anthropology University of Göttingen Göttingen Germany; ^2^ Key Laboratory of Vegetation Ecology, Ministry of Education Northeast Normal University Changchun China; ^3^ Key Laboratory of Urban Environment and Health, Institute of Urban Environment Chinese Academy of Sciences Xiamen China; ^4^ State Key Laboratory of Black Soils Conservation and Utilization, Key Laboratory of Wetland Ecology and Environment, Northeast Institute of Geography and Agroecology Chinese Academy of Sciences Changchun China; ^5^ Centre of Biodiversity and Sustainable Land Use University of Göttingen Göttingen Germany; ^6^ LOEWE Centre for Translational Biodiversity Genomics Frankfurt am Main Germany; ^7^ Senckenberg Biodiversity and Climate Research Centre Frankfurt am Main Germany

**Keywords:** biogeography, geographic range size, mountains, oribatid mites, reproductive mode, trophic niche

## Abstract

Understanding ecological and evolutionary mechanisms that drive biodiversity patterns is important for comprehending biodiversity. Despite being critically important to the functioning of ecosystems, the mechanisms driving belowground biodiversity are little understood. We investigated the radiation and trait diversity of soil oribatid mites from two mountain ranges, that is, the Alps in Austria and Changbai Mountain in China, at similar latitude in the temperate zone differing in formation processes (orogenesis) and exposed to different climates. We collected and sequenced soil oribatid mites from forests at 950–1700 m at each mountain and embedded them into the chronogram of species from temperate Eurasia. We investigated the phylogenetic age of oribatid mites and compared the node age of species with the mountain uplift time of the Alps and Changbai Mountain. We then inspected trophic variation, geographical range size, and reproductive mode, and identified traits that promote oribatid mite survival and evolution in montane forest ecosystems. We found that oribatid mites on Changbai Mountain are phylogenetically older than species in the Alps. All species on Changbai Mountain evolved long before the uplift of Changbai Mountain, but some species in the Alps evolved after the orogenesis of the Alps. On Changbai Mountain, more species possess broader trophic variation, have larger geographical range sizes, and more often reproduce via parthenogenesis compared to species from the Alps. Species on Changbai Mountain survived the mountain uplift or colonized the mountain thereafter, supporting the view that generalistic traits promote survival and evolution in phylogenetically old soil animal species. Collectively, our findings highlight that combining species traits and phylogeny allow deeper insight into the evolutionary forces shaping soil biodiversity in montane ecosystems.

## Graphical abstract

We investigated the radiation and trait diversity of soil oribatid mites from two mountain ranges (the Alps and Changbai Mountain). We found that phylogenetically older oribatid mites of mountain ranges have broader trophic variation, larger geographical range sizes, and more often reproduce via parthenogenesis. Our finding highlights that combining species traits and phylogeny allows deeper insight into the ecological and evolutionary mechanisms shaping soil biodiversity in montane ecosystems.

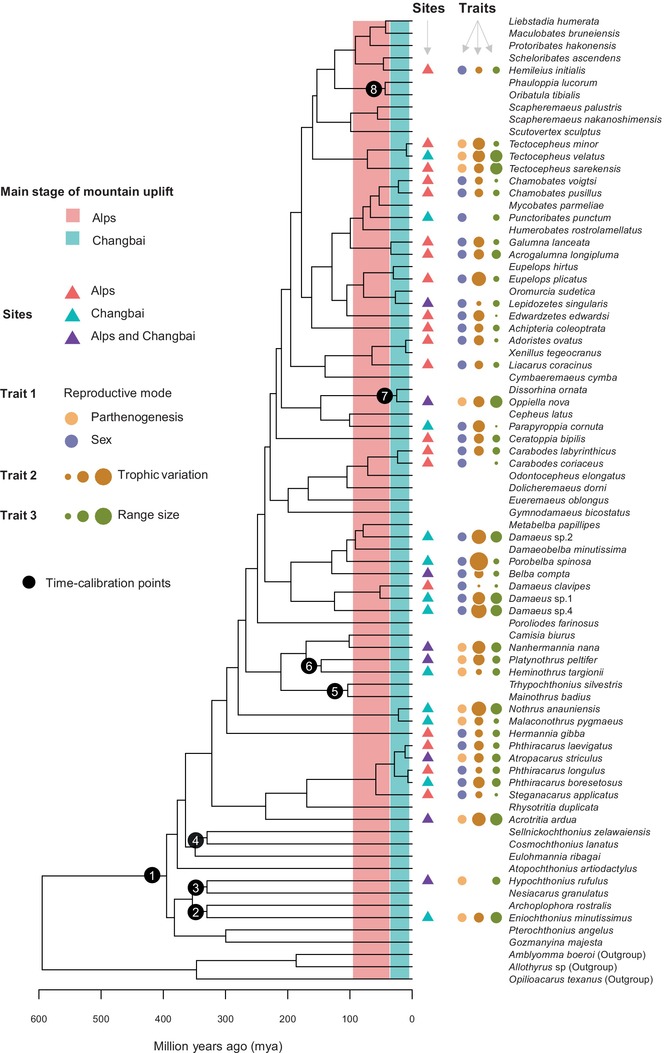

## 1 Introduction

Ecology, biogeography, and phylogeny are fields in biology that are rarely studied in concert (Wiens and Donoghue 2004). On one hand, studies investigating the biogeography of species often ignore their ecology (Wiens 2011). On the other hand, ecologists often neglect that physical and biological barriers (vicariance) and past dispersal events, that is, historical biogeographic factors, affect current patterns of diversity and community structure of animals and plants (Wiens 2011). Integrating ecology, biogeography, and phylogeny is promising since it may allow uncovering how evolutionary processes shape the communities and species distributions of today (Ricklefs and Jenkins 2011).

Although originally based on islands (MacArthur and Wilson 1963, 1967), island biogeography is now increasingly applied to representative insular habitats in mainland systems such as mountains (Costanzi and Steifetten 2019), forests (Bueno and Peres 2019) and lakes (Si et al. 2016). Mountain formation shapes the distribution and biodiversity of organisms since, with orogenesis, new habitats and abiotic environments are formed, increasing habitat heterogeneity (Antonelli et al. 2018; Rahbek et al. 2019). Mountains can also limit the dispersal of species, resulting in the isolation of populations and consequently the evolution of species (Favre et al. 2015). Community assembly along steep environmental gradients and the radiation of species result in mountain regions harboring one‐third of the global terrestrial species, especially being home to a large number of small‐ranged species (Rahbek et al. 2007), despite covering only approximately one‐eighth of the Earth's land surface (Perrigo et al. 2020). In addition, as mountain formation often is dated geologically, community assembly as well as evolutionary processes may be followed and disentangled in detail (Emerson and Gillespie 2008).

The importance of generalism, that is, the ability of species to colonize and maintain populations in diverse habitats, and specialization, that is, the ability to persist only in certain restricted habitats, on macro‐evolutionary patterns has been debated for over a century (Van Tienderen 1991). In recent decades, a growing number of studies tested the hypothesis that specialization in older lineages is an “evolutionary dead end” because it decreases speciation rate, increases extinction rate, and reduces the capacity for future evolutionary change, and vice versa for generalism (Verde Arregoitia et al. 2013; Day et al. 2016; Sriswasdi et al. 2017). Traits of species reflect their evolutionary responses to environmental variations and reflect their ecological strategies (McGill et al. 2006; Violle et al. 2007, 2014; Ottaviani et al. 2020). A subset of functional traits may allow understanding of the differential performance and distribution of species across environmental gradients (Winemiller et al. 2015). For example, the variability of trophic positions, an essential trait related to resource acquisition and utilization, is driven by eco‐evolutionary feedbacks and local environmental conditions (Moosmann et al. 2021). Broad trophic niches allow consumers to exploit a wide range of resources thereby promoting local diversity by reducing extinction probability (Schalk et al. 2017). Further, broad trophic niches also foster the ability of species to colonize wide geographic ranges. Reproductive mode (i.e., sexual vs. asexual reproduction), another key life‐history trait, is tightly related to population dynamics (Juliano 2007). Although sexual reproduction dominates in animal species, many species reproduce asexually (Bell 1982). Two hypotheses, the General‐Purpose‐Genotype (GPG) and the Frozen‐Niche‐Variation (FNV) hypothesis, have been proposed to explain the long‐term persistence of parthenogenetic species (Vrijenhoek and Parker 2009). The General‐Purpose‐Genotype hypothesis views clonal species as generalists and predicts that in the long‐term, asexual species evolve broadly adapted genotypes that are able to tolerate a wide range of environmental factors (Baker 1965; Lynch 1984). By contrast, the Frozen‐Niche‐Variation hypothesis views clonal species as specialists and assumes that asexual species possess “frozen” genotypes from narrowly adapted sexual progenitors (Vrijenhoek 1984; Vrijenhoek and Parker 2009). Consistent with the General‐Purpose‐Genotype hypothesis, asexual species often are geographically widely distributed and colonize high latitudes, high altitudes, islands, and disturbed environments (Peck et al. 1998).

Most studies on the biogeography of montane species have focused on plants and large animals, whereas belowground animal species have received little attention (McCain and Grytnes 2010). Moreover, previous studies on the biogeography of montane soil taxa concentrated on their current habitats (King et al. 2010; Pan et al. 2023a) and species traits (Pan et al. 2023b; Yu et al. 2025), while neglecting the geological history of mountain regions and the evolution process of species (McCain and Grytnes 2010; Antonelli et al. 2018; Xie et al. 2022). Oribatida (Acari: Acariformes) are evolutionary old soil animals that—based on molecular clock estimates—originated in early Paleozoic or even Precambrian times (Schaefer et al. 2010). Today, more than 11,000 species have been described, but overall 100,000 may exist (Subías 2022a; Behan‐Pelletier and Lindo 2023). Notably, there is a good fossil record of oribatid mites ranging from the Devonian to Miocene amber (Krivolutsky and Druk 1986; Sidorchuk 2018). Furthermore, oribatid mites currently occupy virtually any ecosystem in the world, including mountain ranges such as the Alps in Europe and Changbai Mountain in Asia (Pan et al. 2023a). To better understand the assembly processes of oribatid mite communities as well as their biogeographic ranges, recent studies are increasingly using molecular data and traits of species (Pachl et al. 2017; Schaefer and Caruso 2019; Maraun et al. 2022, 2023).

Here, we used two mountain regions to investigate ecological and evolutionary factors structuring oribatid mite communities. These were the Alps in Europe and Changbai Mountain in Asia, differing in formation processes (orogenesis) but located at similar latitudes. We hypothesized that (1) oribatid mites from the Alps are phylogenetically older than those from Changbai Mountain since the formation of the Alps predates that of Changbai Mountain, and (2) phylogenetically older oribatid mite species have broader trophic niches, are more frequently reproducing via parthenogenesis, and are generally more widely distributed than younger species.

## 2 Materials and Methods

### 2.1 Study Sites and Their Orogenesis

**FIGURE 1:**
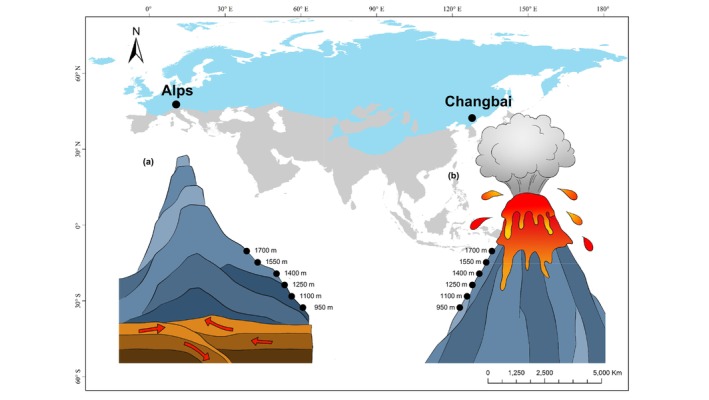
Geographic position, orogenesis, the extend of Quaternary glaciation of the study sites and sampling locations along the studied altitudinal gradients spanning from 950 to 1700 m in the Alps (Austria) and on Changbai Mountain (China). Alps was formed gradually after a series of continental plate collisions (a) while Changbai Mountain was formed gradually after a series of volcanic eruptions (b). The main stage of mountain uplift was 99.6–23.0 mya in the Alps and 22.6–0.01 mya on Changbai Mountain. Both mountains experienced the Quaternary glaciation with the light blue area representing the extent of permafrost in the Eurasia during the Last Permafrost Maximum (Vandenberghe et al. 2014).

The study was carried out in the Alps (43°29′–48°20′ N; 5°2′–16°21′ E) in central Europe and on Changbai Mountain (41°41′–42°51′ N; 127°43′–128°16′ E) in eastern Asia (Figure 1). The Alps and Changbai Mountain differ in range and topology but are located at similar latitude. Alpine orogenesis started in the eastern Austroalpine and ended before the Late Cretaceous, ca. 99–66 million years ago (mya). At the end of the Eocene, ca. 56–34 mya, the plate subduction‐related regime was replaced by magmatic activity, which ceased by the Late Oligocene while plate convergence progressed until ca. 33–23 mya (Piaz et al. 2003). Today, the region is characterized by a relatively warm, temperate oceanic climate. From the bottom at 740 m to the peak in the study region at 2277 m, the mean annual temperature decreases from 6.2°C to 2.1°C, and the annual temperature range is 24°C (Pan et al. 2023a). In the Alps, the vegetation below 1100 m comprises managed beech forests, followed by managed spruce‐beech forests at 1100–1550 m and mountain pine bushes above 1550 m (Wallnöfer and Hotter 2008; Leitinger et al. 2015). The peripheral areas in the Alps typically are dominated by calcareous bedrock (Beniston 2006). The earliest Changbai eruptive episode for the pre‐shield stage is dated from the Late Oligocene to the Early Miocene, ca. 23–10 mya. After the shield‐forming stage (ca. 5.0–1.1 mya) and post‐shield stage (ca. 1.8–0.01 mya), Changbai Mountain went through the final cone‐construction stage (ca. 1.2–0.05 mya) in the late Pleistocene and became roughly what it is now (Zhang et al. 2018). The region is characterized by a cool, temperate continental climate. From the bottom at 530 m to the peak at 2200 m, the mean annual temperature decreases from 2.9°C to −4.8°C, and the annual temperature range is 38°C (Pan et al. 2023a). The vegetation on Changbai Mountain is close to natural and follows a clear altitudinal zonation. Mixed coniferous and broad‐leaved forests dominate at the mountain base (700–1100 m), mixed coniferous forests and sub‐alpine mixed coniferous forests dominate at medium altitudes (i.e., 1100–1500 m and 1500–1800 m, respectively), and birch forests form the upper forest boundary (1800–2100 m) up to the alpine tundra above 2100 m (Sang and Bai 2009; Bai et al. 2011; Shen et al. 2013). Parent rock on Changbai Mountain is mainly basalt (Lan et al. 2021; Pan et al. 2023b). Additionally, both the Alps and Changbai Mountain experienced recent significant climate change events, that is, the Quaternary glaciation (ca. 2.6 mya to present) with the Alps being largely covered by glaciers and Changbai Mountain being dominated by permafrost soils (Zhang et al. 2008; Ivy‐Ochs et al. 2009; Vandenberghe et al. 2014). More details on the study sites and their characteristics are given in Pan et al. (2023a, 2023b).

### 2.2 Sampling, Determination and Traits of Oribatid Mites

At both mountains, oribatid mites were sampled from each of five plots (3 m × 3 m) in September (on Changbai Mountain in 2019 and in the Alps in 2017) at each of the six altitudes, that is, 950, 1100, 1250, 1400, 1550, and 1700 m. The five plots per altitude were spaced by at least 30 m to represent independent local communities, as spatial autocorrelation of soil microarthropods typically vanishes beyond 20–30 m (Keitt et al. 2002; Gutiérrez‐López et al. 2010). From each plot, one randomly selected soil core (diameter 5 cm, depth 10 cm), including the litter layer and 0–10 cm soil depth, was taken. The samples were transported to the laboratory, and animals were extracted using Berlese‐Tullgren funnels over 10 days for Changbai Mountain (Berlese 1905; Tullgren 1918) and using high‐gradient extractors over 7 days for the Alps (Kempson et al. 1963) until the substrate was completely dry. Previous studies indicated that the extraction efficiency of the two methods is similar (Marshall 1972; Petersen and Luxton 1982). Oribatid mites were preserved in ethanol, identified to species level, and their reproductive mode was assigned using literature (Norton et al. 1993; Maraun et al. 2019, 2022). More details on sampling, determination, and reproductive mode of oribatid mites are given in Pan et al. (2023a, 2023b). There is uncertainty in existing classifications of oribatid mites, including the ones of Norton and Behan‐Pelletier (2009) and Subías (2022a), and these classifications are likely to change in future mainly due to the frequent convergent evolution of traits (Cordes et al. 2024). We used the classification of oribatid mites from Subías (2022a) in our study due to its completeness since oribatid mite species from all over the world are included.

Stable isotope ratios of nitrogen (^15^N/^14^N) can be used to estimate the trophic level of consumers, with δ^15^N values being enriched per trophic level by an average of ~3.4‰ (Post 2002; Potapov et al. 2019). We used the standard deviation of litter calibrated δ^15^N values to estimate the trophic variation of oribatid mites as given in Pan et al. (2023b) (Table S1). The geographic range of oribatid mite species was estimated using distribution data from Subías (2022a). Although of coarse resolution, the data provide relatively standardized geographical ranges for all described oribatid mite species and have been used previously in studies on the biogeography of oribatid mites (Maraun et al. 2022; Lu et al. 2024). As in Maraun et al. (2022), the size of geographic regions, such as Holarctic, Palearctic, Subtropical, and Neotropical, was taken from Hawkins and Porter (2001) as well as from internet sources (Table S2). In total, 40 oribatid mite species from the two mountains were included in this study, and information on their reproductive mode, trophic variation, and geographic range was compiled.

### 2.3 Phylogenetic Reconstruction of the Soil Oribatid Mite Species Pool of Eurasian Temperate Zone

We used the 18S rDNA gene for reconstructing the phylogeny of oribatid mites (Schaefer et al. 2010; Pachl et al. 2021). Of the 40 oribatid mite species found, sequences of 14 species were generated for this study and deposited in GenBank (Accession Numbers OR820193–OR820206); 26 sequences were downloaded from NCBI GenBank (https://www.ncbi.nlm.nih.gov/) (Table 1). The pipeline for DNA extraction, amplification, and sequencing of each of the 14 species are described in the Supporting Information.

**TABLE 1:** The oribatid mite species from the Alps and Changbai Mountain and their abundance and 18S sequence information. Infraorder and family are given according to Subías (2022a). Among the 40 oribatid mite species, 28 species were from the Alps and 20 species from Changbai Mountain, whereas eight species (marked by asterisks) were sampled at both. Species which accession number marked by asterisks imply that the sequence was generated in this study.

Mountain	Infraorder	Family	Species name	Abundance	Accession number
Alps	Enarthronota	Hypochthoniidae	*Hypochthonius rufulus* *	1	EF093784
Alps	Mixonomata	Euphthiracaridae	*Acrotritia ardua* *	1	OR820196*
Alps	Mixonomata	Phthiracaridae	*Atropacarus striculus* *	410	EF091416
Alps	Mixonomata	Phthiracaridae	*Phthiracarus laevigatus*	158	OR820194*
Alps	Mixonomata	Phthiracaridae	*Phthiracarus longulus*	24	OR820195*
Alps	Mixonomata	Phthiracaridae	*Steganacarus applicatus*	258	GQ864301
Alps	Desmonomata	Nanhermanniidae	*Nanhermannia nana* *	8	KR081624
Alps	Desmonomata	Crotoniidae	Platynothrus peltifer*	128	EF091422
Alps	Brachypylina	Achipteriidae	*Achipteria coleoptrata*	315	EF091418
Alps	Brachypylina	Galumnidae	*Acrogalumna longipluma*	20	GQ864304
Alps	Brachypylina	Liacaridae	*Adoristes ovatus*	42	GQ864286
Alps	Brachypylina	Damaeidae	*Belba compta* *	6	OR820197*
Alps	Brachypylina	Carabodidae	*Carabodes coriaceus*	3	EF093787
Alps	Brachypylina	Carabodidae	*Carabodes labyrinthicus*	3	KX397629
Alps	Brachypylina	Ceratoppiidae	*Ceratoppia bipilis*	6	EU432204
Alps	Brachypylina	Chamobatidae	*Chamobates pusillus*	37	EU432188
Alps	Brachypylina	Chamobatidae	*Chamobates voigtsi*	164	EU432189
Alps	Brachypylina	Damaeidae	*Damaeus clavipes*	2	KR081607
Alps	Brachypylina	Ceratozetidae	*Edwardzetes edwardsi*	11	MH198178
Alps	Brachypylina	Phenopelopidae	*Eupelops plicatus*	3	EF091419
Alps	Brachypylina	Galumnidae	*Galumna lanceata*	2	KX397630
Alps	Brachypylina	Hemileiidae	*Hemileius initialis*	34	OR820193*
Alps	Brachypylina	Hermanniidae	*Hermannia gibba*	64	EF091426
Alps	Brachypylina	Ceratozetidae	*Lepidozetes singularis* *	2	EU432193
Alps	Brachypylina	Liacaridae	*Liacarus coracinus*	5	KR081619
Alps	Brachypylina	Oppiidae	*Oppiella nova* *	450	KR081626
Alps	Brachypylina	Tectocepheidae	*Tectocepheus minor*	5	EF093778
Alps	Brachypylina	Tectocepheidae	*Tectocepheus sarekensis*	15	EF093776
Changbai	Enarthronota	Eniochthoniidae	*Eniochthonius minutissimus*	151	KR081609
Changbai	Enarthronota	Hypochthoniidae	*Hypochthonius rufulus* *	4	EF093784
Changbai	Mixonomata	Euphthiracaridae	*Acrotritia ardua* *	5	OR820196*
Changbai	Mixonomata	Phthiracaridae	*Atropacarus striculus* *	66	EF091416
Changbai	Mixonomata	Phthiracaridae	*Phthiracarus boresetosus*	28	OR820205*
Changbai	Desmonomata	Crotoniidae	*Heminothrus targionii*	6	OR820201*
Changbai	Desmonomata	Malaconothridae	*Malaconothrus pygmaeus*	33	OR820202*
Changbai	Desmonomata	Nanhermanniidae	*Nanhermannia nana* *	47	KR081624
Changbai	Desmonomata	Nothridae	*Nothrus anauniensis*	22	OR820203*
Changbai	Desmonomata	Crotoniidae	Platynothrus peltifer*	31	EF091422
Changbai	Brachypylina	Damaeidae	*Belba compta* *	10	OR820197*
Changbai	Brachypylina	Damaeidae	*Damaeus* sp.1	9	OR820198*
Changbai	Brachypylina	Damaeidae	*Damaeus* sp.2	11	OR820199*
Changbai	Brachypylina	Damaeidae	*Damaeus* sp.4	21	OR8202008*
Changbai	Brachypylina	Ceratozetidae	*Lepidozetes singularis* *	14	EU432193
Changbai	Brachypylina	Oppiidae	*Oppiella nova* *	621	KR081626
Changbai	Brachypylina	Ceratoppiidae	*Parapyroppia cornuta*	29	OR820204*
Changbai	Brachypylina	Damaeidae	*Porobelba spinosa*	2	OR820206*
Changbai	Brachypylina	Punctoribatidae	*Punctoribates punctum*	1	MH198175
Changbai	Brachypylina	Tectocepheidae	*Tectocepheus velatus*	331	EF093781

To reconstruct the soil oribatid mite species pool of the Eurasian temperate zone and improve the phylogenetic and time credibility of the ultrametric tree, we compiled 18S rDNA sequences of 76 Eurasian oribatid mite species in the temperate zone using the following procedure: (1) We used “Oribatida 18S” as a keyword to download all 18S rDNA sequences of oribatid mite species from NCBI GenBank (data accessed on April 6 2023). This resulted in 210 sequences of species (Table S3, Dataset 1). (2) We kept sequences with a sequence length > 1500 bp, resulting in 204 sequences of species (Table S3, Dataset 2). (3) We added the 14 species sequenced in this study, resulting in 218 sequences of species (Table S3, Dataset 3). Sequences of the other 26 species from the two mountains were also included in Dataset 3. We retrieved information on the geographic distribution of the 218 species based on Subías (2022a). (4) From the 218 sequences, we deleted 29 sequences downloaded from NCBI as they were only ascribed to genus level, resulting in 189 sequences of species (Table S3, Dataset 4). (5) For the 149 sequences downloaded from NCBI among the total 189 species, to improve the reliability and generality of the Eurasian species pool and to improve the credibility of the Eurasian species phylogeny tree, we only kept those species occurring in the temperate and boreal zones of Asia and/or Europe. In parallel, using the MAFFT alignment program (Katoh et al. 2002; Katoh and Standley 2013) implemented in Geneious Prime v2022.2.2 (https://www.geneious.com), we found that sequences of some species were identical, though their species names were different (likely due to taxonomic misidentification). Of these sequences, we kept the ones of species that are widespread and common in Eurasia and rather easy to identify; we assumed that the assignment to species in these species is more credible than in rare and more difficult to identify species. This resulted in the deletion of 106 sequences, leaving 83 sequences in the dataset (Table S3, Dataset 5). (6) From this dataset, we deleted five species (*Alismobates reticulatus*, *Fortuynia rotunda* , *Thalassozetes shimojanai*, *Hydrozetes lacustris* , and *Limnozetes rugosus* ) which occur in aquatic or semiaquatic habitats (Table S3, Dataset 6) leaving only soil living species in the dataset. (7) To improve the accuracy of the phylogenetic tree, we deleted *Collohmannia gigantea* since Collohmanniidae occupy an ambiguous position in oribatid mite phylogeny (Cordes et al. 2024) (Table S3, Dataset 7). (8) Further, we deleted the sequence of *Palaeacarus hystricinus* since no species from the Alps and Changbai Mountain belonged to Palaeosomata, and the phylogenetic tree generated using IQ‐TREE v2.2.2.6 (Minh et al. 2020) that included this species was not trustworthy as indicated by the long branch of this species, although phylogenetic trees with and without this species were the same (Figures S1 and S2). This resulted in the final dataset including 76 sequences of species that cover the taxonomic and phylogenetic diversity of the entire taxon (Table S3, Dataset 8). As in previous studies (Pachl et al. 2012; Schaefer and Caruso 2019), we used three species of Parasitiformes, that is, *Allothyrus* sp. (Allothyridae), *Amblyomma boeroi* (Ixodidae), and *Opilioacarus texanus* (Opilioacaridae), as outgroups and downloaded their 18S rDNA sequences from NCBI (Table S3, Dataset 9).

The final 18S rDNA sequences of the 76 oribatid species and the three outgroups were aligned in Geneious Prime v2022.2.2 using the MAFFT plugin in default mode. To get a general overview of the phylogeny of Eurasian oribatid mites, a Maximum‐Likelihood tree was calculated using IQ TREE v2.2.2.3 (Minh et al. 2020) (Figure S2). Divergence dating using fossils as internal calibrations (Table 2) was performed in BEAST v2.7.5 (Bouckaert et al. 2014; Faurby et al. 2024). Details are described in the Supporting Information.

**TABLE 2:** Taxonomic assignment, geological age of fossil oribatid mites, available 18S sequence from Eurasia oribatid mites in this study, and prior settings used in the molecular clock analysis. Thirteen potential priors with a symbol of ‘\’ were eliminated as they impeded chain convergence and had poor ESS values (< 200). Details are described in the electronic Supporting Information.

Infraorder in Subías (2022a)	Oribatid mite fossil species	Time (mya)	Time mean (mya)	Species (available as 18S sequence)	Mean and offset in BEAST	Reference
Enarthronota	*Carbochthonius antrimensis*	326.4–336	331.2	*Cosmochthonius lanatus*	3; 328	Subías and Arillo 2002
Enarthronota	*Palaeohypochthonius jerami*	326.4–336	331.2	*Hypochthonius rufulus*	3; 328	Subías and Arillo 2002
Enarthronota	*Archaeoplophora bella*	326.4–336	331.2	*Archoplophora rostralis*	3; 328	Subías and Arillo 2002
Holosomata	*Hermannia sellnicki*	40.4–48.6	44.5	*Hermannia gibba*	\	Norton 2006
Holosomata	*Juracarus serratus*	145.5–150.8	148.15	*Platynothrus peltifer*	2; 125	Krivolutsky and Krassilov 1977
Holosomata	*Nothrus vazquezae*	99.6–112	105.8	*Nothrus anauniensis*	\	Arillo et al. 2016
Holosomata	*Trhypochthonius lopezvallei*	99.6–112	105.8	*Trhypochthonius silvestris europaeus*	7; 99	Arillo et al. 2012
Brachypylina	*Achipteria (?) obscura*	145.5–150.8	148.15	*Achipteria coleoptrata*	\	Krivolutsky and Krassilov 1977
Brachypylina	*Dissorhina nuda*	2.59–3.6	3.095	*Dissorhina ornata*	\	Miko 2015
Brachypylina	*Dissorhina paleokrasica*	2.59–3.6	3.095	*Dissorhina ornata*	\	Miko 2015
Brachypylina	*Eremaeus denaius*	23.0–28.4	25.7	*Eueremaeus oblongus*	\	Woolley 1971
Brachypylina	*Hypovertex hispanicus*	99.6–112	105.8	*Scutovertex sculptus*	\	Arillo et al. 2016
Brachypylina	*Jureremus foveolatus*	145.5–150.8	148.15	*Cymbaeremaeus cymba*	\	Krivolutsky and Krassilov 1977
Brachypylina	*Jureremus phippsi*	161.2–164.7	162.95	*Cymbaeremaeus cymba*	\	Selden et al. 2008
Brachypylina	*Liacarus shtanchaevae*	99.6–112	105.8	*Liacarus coracinus*	\	Arillo et al. 2022
Brachypylina	*Neoliodes andreneli*	125–130	127.5	*Poroliodes farinosus*	\	Arillo et al. 2019
Brachypylina	*Oppia hurdi*	23.0–28.4	25.7	*Oppiella nova*	2; 23	Woolley 1971
Brachypylina	*Phauloppia*	40.4–48.6	44.5	*Phauloppia lucorum*	2; 42	O'Dowd et al. 1991
Brachypylina	*Platyliodes sellnicki*	99.6–112	105.8	*Poroliodes farinosus*	\	Arillo et al. 2016
Brachypylina	*Scapheremaeus*	33.9–37.2	35.55	*Scapheremaeus palustris*	\	O'Dowd et al. 1991
Oribatid mites	Oribatid mites	385.3–407	396.15	All 76 oribatid mites	5; 384	Shear et al. 1984

### 2.4 Statistical Analyses

To determine whether oribatid mites from the Alps are phylogenetically older than those from Changbai Mountain, we calculated mean pairwise distance (MPD) based on the time‐calibrated ultrametric tree generated by BEAST (Webb et al. 2002; Bouckaert et al. 2014). We derived the standardized effect size of MPD for the Alps and Changbai Mountain by randomizing the species of the phylogenetic distance matrix 999 times, while keeping the number of species the same as observed in each of the mountains, using the *ses.mpd* function in the R package ‘picante’ (Kembel et al. 2010). Results of observed MPD and randomized MPD (null model) for each mountain were visualized using the *hist* function implemented in ‘graphics’ package. Statistical analyses were carried out using R v4.1.3 with R studio interface (R Core Team 2022). To assess whether most divergence events of species occurred after mountain uplift, we compared the node age of each species with the respective mountain uplift time (the Alps, 99.6–22.3 mya; Changbai Mountain, 22.6–0.01 mya) at different stages (Table S4).

Before testing the trait differences of species from the two mountains, we measured the phylogenetic signal in reproductive mode (sex and parthenogenesis) using the *phylo.d* function in the ‘caper’ package (Fritz and Purvis 2010; Orme et al. 2023), in mean trophic variation, and in mean range size using Blomberg's K and Pagel's lambda implemented in the *phylosig* function in the ‘phytools’ package (Blomberg et al. 2003; Revell 2012). The reproductive mode was phylogenetically conserved; it did not deviate significantly from the Brownian motion model (estimated D = −0.471, probability of E[D] resulting from Brownian phylogenetic structure = 0.846). Blomberg's K and Pagel's lambda were not significant for trophic variation (*K* = 0.266, *p* = 0.33; lambda < 0.01, *p* = 1.00) and range size (*K* = 0.196, *p* = 0.46; lambda < 0.01, *p* = 1.00). We compared the trophic variation and range size using the *wilcox.test* function in the ‘stats’ package since the data were not normally distributed and variances were not homogeneous. To account for the number of measurements in analyzing trophic variation, we also fitted a linear regression model using the *lm* function in the ‘stats’ package; the result indicated that they are not closely correlated (*p* = 0.681, *R* ^2^ < 0.01; Figure S3; Table S1).

## 3 Results

### 3.1 Phylogeny of Oribatid Mites

**FIGURE 2:**
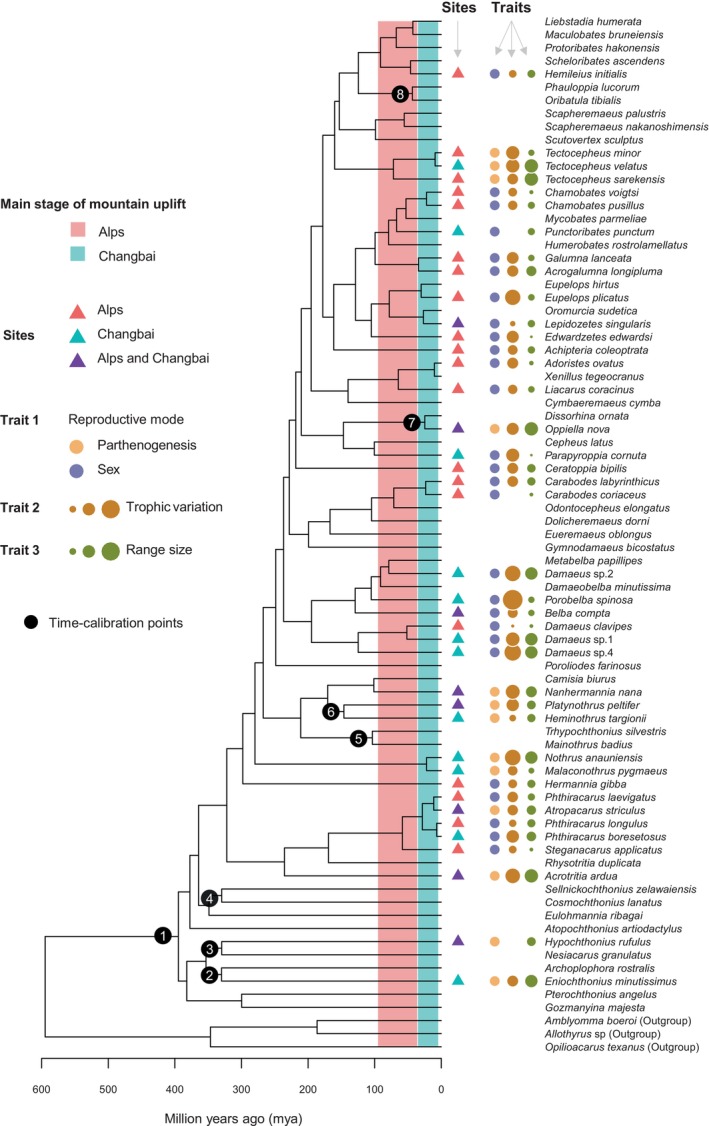
Phylogeny and traits (reproductive mode, trophic variation and range size) of oribatid mites from the Alps (28 species) and Changbai Mountain (20 species), embedded into the chronogram of the oribatid mite species from the Eurasian continent (76 species). *Amblyomma boeroi* , *Allothyrus* sp., and *Opilioacarus texanus* (Acari: Parasitiformes) are used as outgroups. The phylogeny is based on 18S rDNA sequences. Numbers on black circles show the distribution of nodes with species known from the fossil record that were used as priors in the molecular clock analysis ((1) oribatid mites, 407–385 mya, (2) Protoplophoridae, 336–326 mya, (3) Hypochthoniidae, 336–326 mya, (4) Cosmochthoniidae, 336–326 mya, (5) Trhypochthoniidae, 112–99 mya, (6) Trhypochthoniidae, 151–146 mya, (7) Oppiidae, 28–23 mya, (8) Oribatulidae, 49–40 mya; for details and references see Table 2). The red block indicates the main stage of mountain uplift in the Alps (99.6–23.0 mya), the green block indicates the main stage of mountain uplift on the Changbai Mountain (22.6–0.01 mya). The red, green, and purple triangles represent the oribatid mite species in the Alps, on Changbai Mountainand at both of the two mountains, respectively. The orange and blue circles represent parthenogenetic and sexual oribatid mite species, respectively. The brown and green circles represent trophic variation (calculated based on standard deviation in Δ15N values; for details see Section 2 and Table S4) and known geographical range size of oribatid mite species, with the size of circles representing the degree of trophic variation and the size of the geographical range size.

The 76 species compiled for Eurasian oribatid mites, belonging to 65 genera, 42 families, 30 superfamilies, and 4 infraorders, well represent the spectrum of Eurasian oribatid mites and cover the taxonomic diversity of Oribatida (Figure 2; Table S3). Among the 76 oribatid mites, 40 species (belonging to 31 genera, 21 families, 16 superfamilies and 4 infraorders) were collected from the Alps and Changbai Mountain, with 28 species (2177 individuals) from the Alps, 20 species (1442 individuals) from Changbai Mountain, and 8 species from both mountains (Table 1). In the Alps, 20 species (71%) and 1189 individuals (55%) belonged to Brachypylina, while on the Changbai Mountain, 10 species (50%) and 1019 individuals (71%) belonged to Brachypylina. The final tree topology was robust and consistent with previously published phylogenies based on 18S rDNA (Schaefer et al. 2010; Pachl et al. 2017; Maraun et al. 2022) or on more markers (Pepato and Klimov 2015).

**FIGURE 3:**
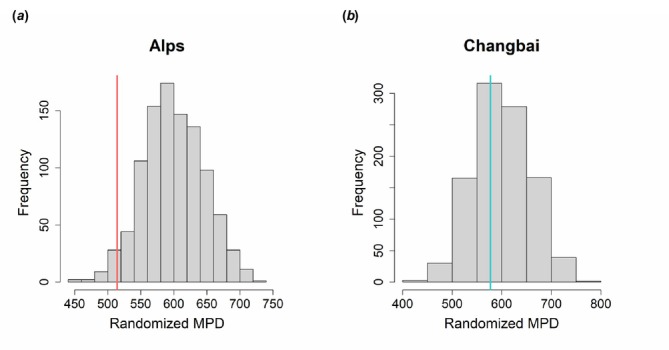
Distribution of the null expectation of randomized mean pairwise distance (MPD) of oribatid mite divergence estimate for the Alps (a) and Changbai Mountain (b). The red and green vertical lines represent the observed MPD for the Alps (514 mya) and Changbai Mountain (577 mya), respectively; for details see text.

Generally, species from Changbai Mountain were phylogenetically older than those from the Alps. This was further supported by the standardized effect size of MPD of oribatid mites from the Alps and Changbai Mountain (Figure 3). For species in the Alps, the observed MPD (514 mya) was significantly younger than the expectation (600 my; mean randomized MPD from the null model) (*mpd.obs.z* = −1.92, *mpd.obs.p* < 0.025; Figure 3a). For species on Changbai Mountain, the observed MPD (577 mya) was not significantly different from the expectation (601 my; mean randomized MPD from the null model) (*mpd.obs.z* = −0.40, *mpd.obs.p* > 0.025; Figure 3b). The mean randomized MPD of oribatid mites from both mountains was about 600 my, with that from Changbai (577 mya) exceeding that from the Alps (514 mya) indicating that the latter are phylogenetically younger (Figure 3).

### 3.2 Divergence Time and Mountain Uplift

There were no species that diverged on Changbai Mountain after the latest eruptive episode (0.01 mya) in the Holocene, whereas 18% of the oribatid mite species in the Alps diverged after the latest Alpine orogenesis in the Miocene (23 mya) (Figure 2; Figure S4; Table S4).

### 3.3 Trait Composition

**FIGURE 4:**
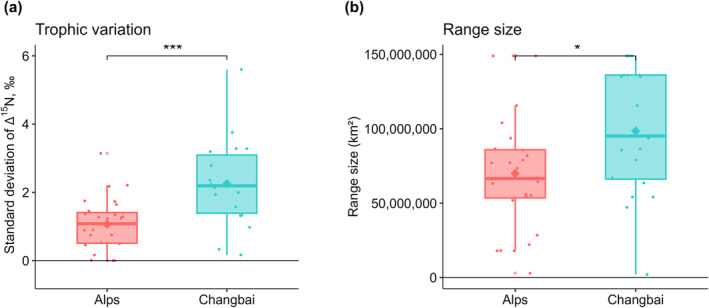
Trophic variation (a) and geographical range size (b) of oribatid mite species from the Alps and Changbai Mountain. The box plots include mean (rhombic symbol), median (horizontal line), and interquartile range (box); error bars represent 95% confidence intervals. Each point represents one species. Asterisks indicate significant effects, with *p < 0.05 and ***p < 0.001.

Among the 28 species in the Alps, 8 (29%) were parthenogenetic, whereas on Changbai Mountain, 11 of the 20 species (55%) were parthenogenetic. Trophic variation and geographic range size of oribatid mite species on Changbai Mountain were significantly higher than in the Alps (*W* = 96, *p* < 0.001 and *W* = 96, *p* < 0.05, respectively; Figure 4). On average, trophic variation as measured by variations in Δ^15^N values in the Alps was 1.07‰ ± 0.76‰, and on Changbai Mountain, it was 2.25‰ ± 1.30‰ (Figure 4a; Table S1); the mean range size of oribatid mite species in the Alps was 70,035,220 ± 39,097,524 km^2^, and that of oribatid mite species on Changbai Mountain was 98,501,210 ± 42,053,334 km^2^ (Figure 4b; Table S5).

## 4 Discussion

It is challenging to disentangle whether the current pattern of species diversity results from adaptation to local conditions, phenotypic plasticity, or both (Ackerly 2003; Vellend 2010, 2016). Mountain biodiversity is particularly suited to disentangle ecological and evolutionary processes contributing to local biodiversity (Rahbek et al. 2019). We found that species from the Changbai Mountain were phylogenetically older than those from the Alps, contradicting our first hypothesis. Specifically, all oribatid mite species existed long before the uplift of Changbai Mountain, whereas in the Alps, 18% of the species evolved after the mountain uplift. Species traits, that is, trophic variation and range size, did not exhibit phylogenetic signal, suggesting that these traits are evolutionarily labile and that ecological processes dominate in structuring the current oribatid mite communities in both mountain regions. Furthermore, in line with our second hypothesis, our results suggest that each of the three traits studied contribute to the persistence of evolutionary old species. In the following, we discuss the contribution of evolutionary processes and generalistic traits to the long‐term survival of oribatid mites.

### 4.1 Evolution and Radiation of Species

The oribatid mite species from the Alps were on average phylogenetically younger than the species from Changbai Mountain, which contrasts with our first hypothesis. In the Alps, 18% of the oribatid mite species diverged after the latest Alpine orogeny (ca. 23 mya) in the Miocene, and most species (71%) and individuals (55%) were derived Brachypylina, indicating that mountain uplift may accelerate the radiation of species (Hughes and Eastwood 2006; Schmitt 2009; Wang et al. 2022). The lower likelihood ratio and bootstrap approximation for species in the Alps than for those on Changbai Mountain also point to rapid radiation (Whitfield and Lockhart 2007; Whitfield and Kjer 2008). By contrast, the oribatid mite species on Changbai Mountain that we included in the phylogenetic study uniformly evolved prior to the mountain uplift (ca. 22.6–0.01 mya) and did not radiate afterwards. Similar results were also found in other soil animal species (Collembola) on Changbai Mountain (Xie et al. 2022). Since Changbai Mountain is much younger than the Alps, the time for diversification may not have been long enough, which is consistent with the very slow speciation in oribatid mites and soil animals in general (Marshall and Pugh 1996). Additionally, all oribatid mite species from the Alps and Changbai Mountain included in this study survived the Quaternary glaciation, emphasizing the role of for example, nunataks, peripheral, and lowland refugia, in mountains serving as glacial refuges for soil animals to survive long evolutionary time scales (Holderegger and Thiel‐Egenter 2009; Lohse et al. 2011; Brighenti et al. 2021).

### 4.2 Traits Contributing to Long‐Term Survival of Species

Trophic variation is based on both environmental and genetic determinants (Moosmann et al. 2021). Trophic variation of the studied oribatid mite species did not show phylogenetic signal, suggesting that it is evolutionarily labile and mainly determined by environmental conditions (Webb et al. 2002; Swenson 2019). As indicated by variations in Δ^15^N values, trophic variation of oribatid mite species on Changbai Mountain was significantly higher than in the Alps, suggesting that the phylogenetically older species on Changbai Mountain are characterized by wider trophic niches, indicating more dietary flexibility (Cirtwill et al. 2018). For example, *Porobelba spinosa* (Δ^15^N = 9.2‰ ± 5.6‰) and *Parapyroppia cornuta* (Δ^15^N = 5.6‰ ± 2.1‰) from Changbai Mountain are likely to live as predators/scavengers, presumably feeding on nematodes, enchytraeids, eggs of other invertebrates, and carcasses of other animals (Heidemann et al. 2011; Maraun et al. 2023; Pan et al. 2023b). When such prey is in short supply, they may turn to feed on fungi. Further, *Nothrus anauniensis* (Δ^15^N = 3.9‰ ± 3.3‰), *Acrotritia ardua* (Δ^15^N = 2.2‰ ± 2.8‰), and *Nanhermannia nana* (Δ^15^N = 3.2‰ ± 3.3‰) from Changbai Mountain likely live as secondary decomposers, mainly feeding on fungi, but change their diet to litter when fungi are in short supply (Pan et al. 2023b).

We found the proportion of parthenogenetic species on Changbai Mountain to be higher than in the Alps, supporting earlier findings that phylogenetically old species of oribatid mites are more often parthenogenetic (Pachl et al. 2021). Generally, the findings support the General‐Purpose‐Genotype hypothesis and support the assumption that broad‐adapted general genotypes in phylogenetically old parthenogenetic species allow them to cope with environmental changes, while specialized genotypes vanish in the long term due to changes in environmental conditions in space and time (Baker 1965; Lynch 1984). Our finding that parthenogenetic species are phylogenetically older than sexual species in mountain ranges argues against the Frozen‐Niche‐Variation hypothesis, proposing that the recurrent freezing of new clonal genotypes from extant sexual ancestors allows asexual species to refresh their genotypes (Vrijenhoek and Parker 2009), resulting in parthenogenetic species typically being relatively young (Strasburg and Kearney 2005; Hörandl 2009). Additionally, as parthenogenetic species only need a single individual for reproduction, they typically colonize new habitats faster than sexual species and often have broader geographic distribution (Chahartaghi et al. 2009; Hörandl 2009; Maraun et al. 2022).

Species inhabiting larger geographical ranges are less vulnerable to climate change and disturbances (Angert et al. 2011), resulting in the range size of species and extinction risk correlated negatively (Birand et al. 2012). The average geographic range size of the phylogenetically older oribatid mite species on Changbai Mountain considerably exceeded that of species from the Alps, coinciding with a recent study finding positive species age–range size relationships in four terrestrial vertebrate groups (birds, mammals, non‐avian reptiles, and amphibians) (Guo et al. 2024), and supporting the assumption that species with large ranges are less vulnerable to extinction. The larger range size of species on Changbai Mountain than in the Alps likely also reflects that the species on Changbai Mountain tend to be more generalistic and more variable in their diet (Lanszki et al. 2022), and have a larger fraction of parthenogenetic species (having larger range sizes than sexual species) as discussed above (Birand et al. 2012; Maraun et al. 2022). By contrast, species with small ranges in the Alps tended to be locally rare, likely reflecting that they are more specialized (Gaston and Blackburn 2000), with both factors increasing their extinction risk (Davies et al. 2004; Böhm et al. 2016). Additionally, widely distributed species typically are locally abundant, and this applied for example, to *Nothrus anauniensis* and *Eniochthonius minutissimus* on Changbai Mountain, reaching densities of > 2000 ind./m^2^ (Pan et al. 2023a).

### 4.3 Limitations and Outlooks

Overall oribatid mite diversity was relatively low, but for example, the low number of species of Oppiidae and Suctobelbidae is consistent with previous studies in the Alps (Fischer et al. 2010) and on Changbai Mountain (Lin et al. 2023; Liu et al. 2023). However, more species of these taxa may occur at higher altitudes (Fischer and Schatz 2013). Comprehensive and accurate trait data in oribatid mite species is another problem which needs further attention, in particular the geographic range size. We estimated distribution data from Subías (2022a) and this data in part has a coarse resolution. Further, the lack of data on the distribution range of the three unknown *Damaeus* species on Changbai Mountain may have affected our conclusions. To improve the results in future studies, a more comprehensive and detailed database on the distribution of oribatid mite species is needed. Additionally, considering the uncertainty of the current classification systems and phylogeny of oribatid mites, for example, if Malaconothroidea is a basal nothrine taxon (Cordes et al. 2024; Norton and Ermilov 2024), and the limited availability of molecular data, we call for more high‐quality molecular data and complete genomes of oribatid mites (Arribas et al. 2020; Ban et al. 2022; Du et al. 2024; Yu et al. 2024). Therefore, our findings, accessed by combining a more reliable phylogeny and accurate trait information, will be refined in the near future.

## 5 Conclusion

Our results show that evolutionary, historical, and ecological processes shape the diversity of contemporary oribatid mite communities. The results highlight the remarkable constancy of soil animal species despite major geological changes, with only excessive periods of time after mountain uplift resulting in the evolution of new soil animal species and increased local soil animal diversity. Further, the results indicate that high trophic variation, parthenogenetic reproduction, and wide geographic distribution facilitates phylogenetically old soil animal species to cope with changing environmental conditions over evolutionary periods of time. Future studies linking historical events, shifts in environmental conditions, and (more) traits in different soil arthropod species may help to disentangle driving factors of mountain species diversity, which is of particular importance in the face of global change.

## Author Contributions

**Xue Pan:** conceptualization (equal), data curation (lead), formal analysis (lead), funding acquisition (equal), investigation (equal), methodology (lead), software (lead), visualization (lead), writing – original draft (lead), writing – review and editing (lead). **Bastian Heimburger:** methodology (equal), software (equal), writing – review and editing (equal). **Ting‐Wen Chen:** methodology (equal), software (equal), writing – review and editing (equal). **Jing‐Zhong Lu:** data curation (equal), methodology (equal), writing – review and editing (equal). **Peter Hans Cordes:** data curation (equal), methodology (equal), writing – review and editing (equal). **Zhijing Xie:** investigation (equal), writing – review and editing (equal). **Xin Sun:** funding acquisition (lead), investigation (lead), project administration (lead), writing – review and editing (equal). **Dong Liu:** methodology (equal), writing – review and editing (equal). **Donghui Wu:** funding acquisition (lead), investigation (lead), project administration (lead), writing – review and editing (equal). **Stefan Scheu:** conceptualization (equal), funding acquisition (lead), investigation (equal), project administration (lead), supervision (equal), writing – review and editing (equal). **Ina Schaefer:** formal analysis (equal), methodology (equal), software (equal), visualization (equal), writing – review and editing (equal). **Mark Maraun:** conceptualization (lead), formal analysis (equal), funding acquisition (equal), methodology (equal), project administration (equal), supervision (lead), writing – review and editing (lead).

## Conflicts of Interest

The authors declare no conflicts of interest.

## Supporting information


Data S1.

Data S2.

Figure S1.

Table S1.

Table S2.

Table S3.

Table S4.

Table S5.

## Data Availability

All data generated or analyzed during this study, original scripts, and Supporting Information are uploaded in the Supporting Information and are available from an open digital repository (Dryad, https://doi.org/10.5061/dryad.2bvq83bz0).
