# Supplementary material for: Older Lineages of Oribatid Mites in Mountain Ranges Have Broader Geographic Ranges and Exhibit More Generalistic Traits
Source: Ecol Evol. 2025 Feb 28;15(3):e71046. doi: 10.1002/ece3.71046 (PMC11871110; doi:10.1002/ece3.71046)
Supplement: Supplementary file 1 — Data S1. [file ECE3-15-e71046-s003.docx]

**Supplementary materials for “Materials and Methods” in**

**Older lineages of oribatid mites in mountain ranges have broader geographic ranges and exhibit more generalistic traits**

**(a) DNA extraction, PCR and sequencing of oribatid mites from the two mountains**

Template DNA of each of the 14 species (among them nine species from the Changbai Mountain, 3 species from the Alps, and 2 species occurring on both mountains; Table 1) was extracted from single individuals using the DNeasy Blood and Tissue Kit (Qiagen, Hilden, Germany) with silica membrane columns and protease K from Genaxxon (25 mM; Genaxxon BioScience, Ulm, Germany) following the manufacturer’s protocol with final elution in 40 µl elution buffer and stored at -20 °C. The MasterMix for a single PCR reaction of the 18S rDNA gen-fragment consisted of 25 µl; containing 4 µl H_2_O, 1.5 µl MgCl_2_, 1 µl of each primer (forward and reverse, each 10 pMol), 12.5 µl SuperHot Taq MasterMix (Genaxxon BioScience, Ulm, Germany) and 5 µl template DNA. Primers for 18S rDNA PCR were 5′-TAC CTG GTT GAT CCT GCC AG-3′ (forward) and 5′-AAT GAT CCT TCC GCA GGT TCA C-3′ (reverse) (Domes *et al.*, 2007). The PCR protocol consisted of an initial activation step at 95 °C for 15 min, 35 amplification cycles (denaturation, 95 °C for 45 s; annealing, 57 °C for 60 s; elongation, 72 °C for 60 s) and a final elongation step at 72 °C for 10 min. All PCR products were visualized on a 1 % agarose gel, purified with the PCR DNA Purification Mini Spin Column Kit (Genaxxon Bioscience), measured the DNA concentration with NanoDrop 2000/2000C v1.6 (Thermo Fisher Scientific Inc.), sequenced at Göttingen Genomics Laboratory (Institute of Microbiology and Genetics, University of Göttingen, Germany), using the additional sequencing primers 18S554f 5′-AAG TCT GGT GCC AGC AGC CGC-3′, 18S1282r 5′-TCA CTC CAC CAA CTA AGA ACG GC-3′, 18S1150f 5′-ATT GAC GGA AGG GCA CCA CCA G-3′ and 18S614r 5′-TCC AAC TAC GAG CTT TTT AAC C-3′ (Domes *et al.*, 2007).

**(b) Phylogeny of the all representative oribatid mites in Eurasia**

**(i) Sequence alignment**

Sequences of 18S rDNA generated for this study were assembled and ambiguous positions were corrected using the chromatograms in Geneious Prime v2022.2.2. A preliminary alignment (2259 bp) including 76 oribatid mite species and three outgroups was generated using MAFF align function in Geneious Prime v2022.2.2. To eliminate poorly aligned positions and divergent regions of the alignment, the final alignment (1490 bp) of 79 sequences for downstream analyses was shortened based on the preliminary alignment using the Gblocks 0.91b with ‘allow gap positions within the final blocks’ option (Talavera & Castresana, 2007).

**(ii) Phylogeny reconstruction**

One tree for general overview was calculated using Maximum Likelihood (ML) algorithms in IQ-TREE v2.2.2.6 (Minh *et al.*, 2020). We used the implemented ModelFinder to determine the best-fit substitution model and chose the model (GTR+F+I+R3) that minimizes the Akaike Information Criterion (AIC) score (Kalyaanamoorthy *et al.*, 2017). We performed the SH-like approximate likelihood ratio test (SH-aLRT; ‘–alrt’ command) (Guindon *et al.*, 2010) and ultrafast bootstrap approximation (UFBoot; ‘–B’ command) (Minh *et al.*, 2013; Hoang *et al.*, 2018) each with 1000 replicates, along with the ‘–bnni’ command to reduce the risk of overestimating branch supports with UFBoot due to severe model violations. Most nodes were well-supported by SH-aLRT values and UFboot values (SH-aLRT >= 80% and UFboot >= 95%; Figure S1).

**(iii) Molecular divergence time estimation**

We estimated divergence times using Bayesian Inference in BEAST v2.7.5 (Bouckaert *et al.*, 2014, 2019). We used the GTR site model with estimate substitution rate, the optimized relaxed clock with mean clock rate as 0.0001, the Yule tree model with exponential priors, the MCMC chain with 80 million generations and a sampling frequency of 4000. We tested combinations of 21 potential priors from the fossil record, and eliminated 13 priors that impeded chain convergence and had poor ESS values (< 200), resulting in eight informative priors for calibration of the phylogenetic tree (Table S3). Chain convergence was checked on Tracer v.1.7.2 (Rambaut *et al.*, 2018) and the final maximum-clade-credibility tree was generated with TreeAnnotator v.2.7.5 using a burn-in of 25 % and a posterior probability limit of 0.8.

**References**

Bouckaert, R., Heled, J., Kühnert, D., Vaughan, T., Wu, C.-H., Xie, D., Suchard, M.A., Rambaut, A. & Drummond, A.J. (2014) BEAST 2: a software platform for Bayesian evolutionary analysis. *PLoS computational biology*, **10**, e1003537.

Bouckaert, R., Vaughan, T.G., Barido-Sottani, J., Duchêne, S., Fourment, M., Gavryushkina, A., Heled, J., Jones, G., Kühnert, D., De Maio, N., & others (2019) BEAST 2.5: An advanced software platform for Bayesian evolutionary analysis. *PLoS computational biology*, **15**, e1006650.

Domes, K., Norton, R.A., Maraun, M. & Scheu, S. (2007) Reevolution of sexuality breaks Dollo’s law. *Proceedings of the National Academy of Sciences of the United States of America*, **104**, 7139–7144.

Guindon, S., Dufayard, J.-F., Lefort, V., Anisimova, M., Hordijk, W. & Gascuel, O. (2010) New algorithms and methods to estimate maximum-likelihood phylogenies: assessing the performance of PhyML 3.0. *Systematic biology*, **59**, 307–321.

Hoang, D.T., Chernomor, O., Von Haeseler, A., Minh, B.Q. & Vinh, L.S. (2018) UFBoot2: improving the ultrafast bootstrap approximation. *Molecular biology and evolution*, **35**, 518–522.

Kalyaanamoorthy, S., Minh, B.Q., Wong, T.K., Von Haeseler, A. & Jermiin, L.S. (2017) ModelFinder: fast model selection for accurate phylogenetic estimates. *Nature methods*, **14**, 587–589.

Minh, B.Q., Nguyen, M.A.T. & Von Haeseler, A. (2013) Ultrafast approximation for phylogenetic bootstrap. *Molecular biology and evolution*, **30**, 1188–1195.

Minh, B.Q., Schmidt, H.A., Chernomor, O., Schrempf, D., Woodhams, M.D., Von Haeseler, A. & Lanfear, R. (2020) IQ-TREE 2: new models and efficient methods for phylogenetic inference in the genomic era. *Molecular biology and evolution*, **37**, 1530–1534.

Rambaut, A., Drummond, A.J., Xie, D., Baele, G. & Suchard, M.A. (2018) Posterior summarization in Bayesian phylogenetics using Tracer 1.7. *Systematic biology*, **67**, 901–904.

Talavera, G. & Castresana, J. (2007) Improvement of phylogenies after removing divergent and ambiguously aligned blocks from protein sequence alignments. *Systematic biology*, **56**, 564–577.
