# Supplementary material for: Older Lineages of Oribatid Mites in Mountain Ranges Have Broader Geographic Ranges and Exhibit More Generalistic Traits
Source: Ecol Evol. 2025 Feb 28;15(3):e71046. doi: 10.1002/ece3.71046 (PMC11871110; doi:10.1002/ece3.71046)
Supplement: Supplementary file 3 — Figure S1. [file ECE3-15-e71046-s002.pdf]

Supplementary materials

Figure S1. The phylogenetic tree includes the *Palaeacarus hystricinus* and was generated based on Maximum Likelihood (ML) as implemented in IQ-TREE v2.2.2.6.

Figure S2. Phylogenetic tree of 76 Eurasian oribatid mite species (excluding *Palaeacarus hystricinus*) based on Maximum Likelihood (ML) as implemented in IQ-TREE v2.2.2.6.

Figure S3. Correlation between the standard deviation of $\Delta^{15}N$ values (i.e., trophic variation) of oribatid mite species and the number of measurements per species from the Alps and Changbai Mountain. Black dashed line denotes overall model and colored lines indicate models for the Alps (red) and for Changbai Mountain (green).

Figure S4. BEAST phylogeny of the 76 Eurasian oribatid mite species with estimated divergence times based on eight fossil constraints. For calibration points and other details see Figure 1 and Table 2. Blue bars indicate 95% credible intervals of the divergence time estimates. Tip labels in red, green, amaranth and black represent the oribatid mites from the Alps, Changbai Mountain and both mountains, respectively.

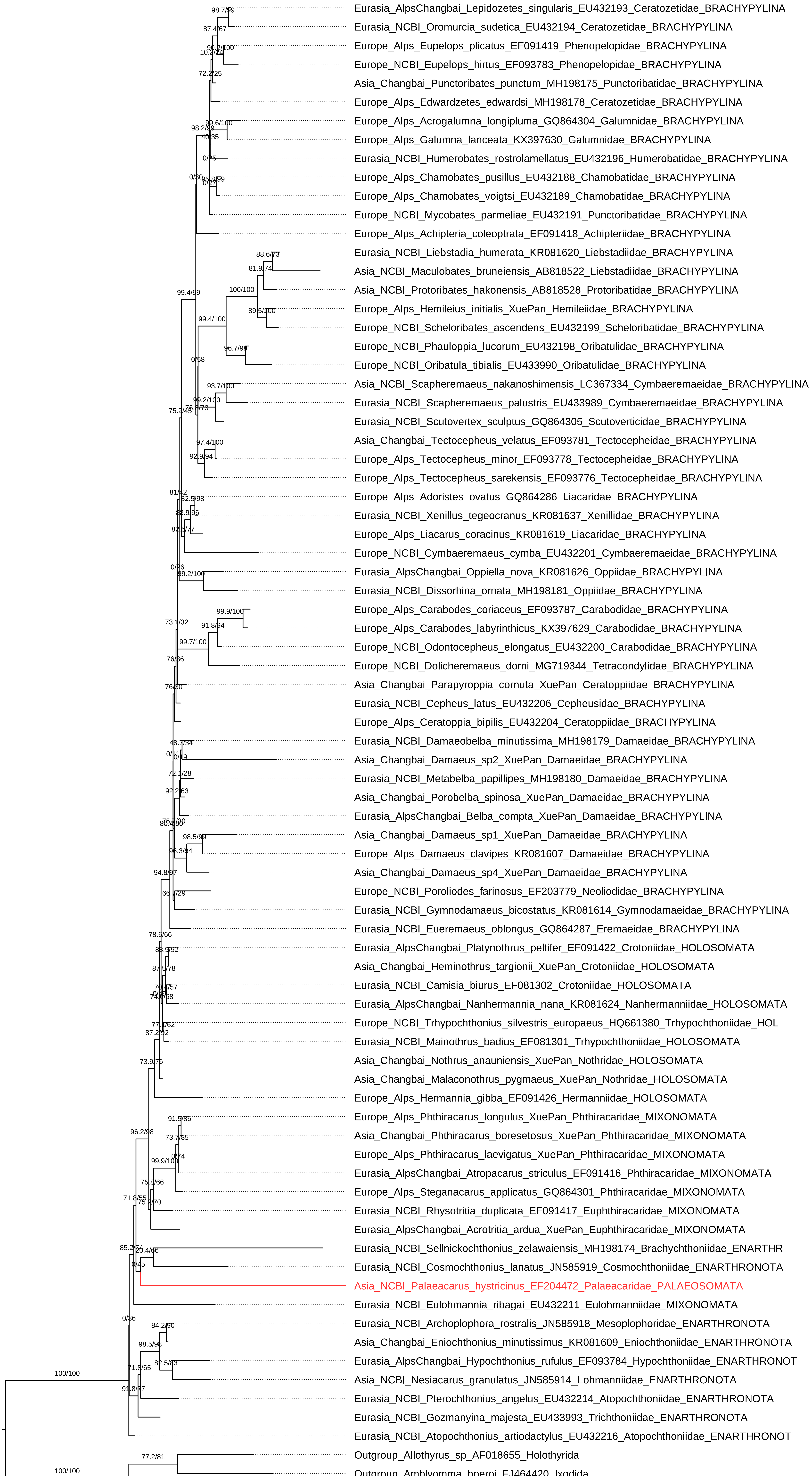
Eurasia_AlpsChangbai_Lepidozetes_singularis_EU432193_Ceratozetidae_BRACHYPYLINA
Eurasia_NCBI_Oromurcia_sudetica_EU432194_Ceratozetidae_BRACHYPYLINA
Europe_Alps_Eupelops_plicatus_EF091419_Phenopelopidae_BRACHYPYLINA
Europe_NCBI_Eupelops_hirtus_EF093783_Phenopelopidae_BRACHYPYLINA
Asia_Changbai_Punctoribates_punctum_MH198175_Punctoribatidae_BRACHYPYLINA
Europe_Alps_Edwardzetes_edwardsi_MH198178_Ceratozetidae_BRACHYPYLINA
Europe_Alps_Acrogalumna_longipluma_GQ864304_Galumnidae_BRACHYPYLINA
Europe_Alps_Galumna_lanceata_KX397630_Galumnidae_BRACHYPYLINA
Eurasia_NCBI_Humerobates_rostrolamellatus_EU432196_Humerobatidae_BRACHYPYLINA
Europe_Alps_Chamobates_pusillus_EU432188_Chamobatidae_BRACHYPYLINA
Europe_Alps_Chamobates_voigtsi_EU432189_Chamobatidae_BRACHYPYLINA
Europe_NCBI_Mycobates_parmeliae_EU432191_Punctoribatidae_BRACHYPYLINA
Europe_Alps_Achipteria_coleoptrata_EF091418_Achipteriidae_BRACHYPYLINA
Eurasia_NCBI_Liebstadia_humerata_KR081620_Liebstadiidae_BRACHYPYLINA
Asia_NCBI_Maculobates_bruneiensis_AB818522_Liebstadiidae_BRACHYPYLINA
Asia_NCBI_Protoribates_hakonensis_AB818528_Protoribatidae_BRACHYPYLINA
Europe_Alps_Hemileius_initialis_XuePan_Hemileiidae_BRACHYPYLINA
Europe_NCBI_Scheloribates_ascendens_EU432199_Scheloribatidae_BRACHYPYLINA
Europe_NCBI_Phauloppia_lucorum_EU432198_Oribatulidae_BRACHYPYLINA
Europe_NCBI_Oribatula_tibialis_EU433990_Oribatulidae_BRACHYPYLINA
Asia_NCBI_Scapheremaeus_nakanoshimensis_LC367334_Cymbaeremaeidae_BRACHYPYLINA
Eurasia_NCBI_Scapheremaeus_palustris_EU433989_Cymbaeremaeidae_BRACHYPYLINA
Eurasia_NCBI_Scutovertex_sculptus_GQ864305_Scutoverticidae_BRACHYPYLINA
Asia_Changbai_Tectocepheus_velatus_EF093781_Tectocepheidae_BRACHYPYLINA
Europe_Alps_Tectocepheus_minor_EF093778_Tectocepheidae_BRACHYPYLINA
Europe_Alps_Tectocepheus_sarekensis_EF093776_Tectocepheidae_BRACHYPYLINA
Europe_Alps_Adoristes_ovatus_GQ864286_Liacaridae_BRACHYPYLINA
Eurasia_NCBI_Xenillus_tegeocranus_KR081637_Xenillidae_BRACHYPYLINA
Europe_Alps_Liacarus_coracinus_KR081619_Liacaridae_BRACHYPYLINA
Europe_NCBI_Cymbaeremaeus_cymba_EU432201_Cymbaeremaeidae_BRACHYPYLINA
Eurasia_AlpsChangbai_Oppiella_nova_KR081626_Oppiidae_BRACHYPYLINA
Eurasia_NCBI_Dissorhina_ornata_MH198181_Oppiidae_BRACHYPYLINA
Europe_Alps_Carabodes_coriaceus_EF093787_Carabodidae_BRACHYPYLINA
Europe_Alps_Carabodes_labyrinthicus_KX397629_Carabodidae_BRACHYPYLINA
Europe_NCBI_Odontocepheus_elongatus_EU432200_Carabodidae_BRACHYPYLINA
Europe_NCBI_Dolicheremaeus_dorni_MG719344_Tetracondylidae_BRACHYPYLINA
Asia_Changbai_Parapyroppia_cornuta_XuePan_Ceratoppiidae_BRACHYPYLINA
Eurasia_NCBI_Cepheus_latus_EU432206_Cepheusidae_BRACHYPYLINA
Europe_Alps_Ceratoppia_bipilis_EU432204_Ceratoppiidae_BRACHYPYLINA
Eurasia_NCBI_Damaeobelba_minutissima_MH198179_Damaeidae_BRACHYPYLINA
Asia_Changbai_Damaeus_sp2_XuePan_Damaeidae_BRACHYPYLINA
Eurasia_NCBI_Metabelba_papillipes_MH198180_Damaeidae_BRACHYPYLINA
Asia_Changbai_Porobelba_spinosa_XuePan_Damaeidae_BRACHYPYLINA
Eurasia_AlpsChangbai_Belba_compta_XuePan_Damaeidae_BRACHYPYLINA
Asia_Changbai_Damaeus_sp1_XuePan_Damaeidae_BRACHYPYLINA
Europe_Alps_Damaeus_clavipes_KR081607_Damaeidae_BRACHYPYLINA
Asia_Changbai_Damaeus_sp4_XuePan_Damaeidae_BRACHYPYLINA
Europe_NCBI_Poroliodes_farinosus_EF203779_Neoliodidae_BRACHYPYLINA
Eurasia_NCBI_Gymnodamaeus_bicostatus_KR081614_Gymnodamaeidae_BRACHYPYLINA
Eurasia_NCBI_Eueremaeus_oblongus_GQ864287_Eremaeidae_BRACHYPYLINA
Eurasia_AlpsChangbai_Platynothrus_peltifer_EF091422_Crotoniidae_HOLOSOMATA
Asia_Changbai_Heminothrus_targionii_XuePan_Crotoniidae_HOLOSOMATA
Eurasia_NCBI_Camisia_biurus_EF081302_Crotoniidae_HOLOSOMATA
Eurasia_AlpsChangbai_Nanhermannia_nana_KR081624_Nanhermanniidae_HOLOSOMATA
Europe_NCBI_Trhypochthonius_silvestris_europaeus_HQ661380_Trhypochthoniidae_HOL
Eurasia_NCBI_Mainothrus_badius_EF081301_Trhypochthoniidae_HOLOSOMATA
Asia_Changbai_Nothrus_anauniensis_XuePan_Nothridae_HOLOSOMATA
Asia_Changbai_Malaconothrus_pygmaeus_XuePan_Nothridae_HOLOSOMATA
Europe_Alps_Hermannia_gibba_EF091426_Hermanniidae_HOLOSOMATA
Europe_Alps_Phthiracarus_longulus_XuePan_Phthiracaridae_MIXONOMATA
Asia_Changbai_Phthiracarus_boresetosus_XuePan_Phthiracaridae_MIXONOMATA
Europe_Alps_Phthiracarus_laevigatus_XuePan_Phthiracaridae_MIXONOMATA
Eurasia_AlpsChangbai_Atropacarus_striculus_EF091416_Phthiracaridae_MIXONOMATA
Europe_Alps_Steganacarus_applicatus_GQ864301_Phthiracaridae_MIXONOMATA
Eurasia_NCBI_Rhysotritia_duplicata_EF091417_Euphthiracaridae_MIXONOMATA
Eurasia_AlpsChangbai_Acrotritia_ardua_XuePan_Euphthiracaridae_MIXONOMATA
Eurasia_NCBI_Sellnickochthonius_zelawaiensis_MH198174_Brachychthoniidae_ENARTHR
Eurasia_NCBI_Cosmochthonius_lanatus_JN585919_Cosmochthoniidae_ENARTHRONOTA
Asia_NCBI_Palaeacarus_hystricinus_EF204472_Palaeacaridae_PALAEOSOMATA
Eurasia_NCBI_Eulohmannia_ribagai_EU432211_Eulohmanniidae_MIXONOMATA
Eurasia_NCBI_Archoplophora_rostralis_JN585918_Mesoplophoridae_ENARTHRONOTA
Asia_Changbai_Eniochthonius_minutissimus_KR081609_Eniochthoniidae_ENARTHRONOTA
Eurasia_AlpsChangbai_Hypochthonius_rufulus_EF093784_Hypochthoniidae_ENARTHRONOT
Asia_NCBI_Nesiacarus_granulatus_JN585914_Lohmanniidae_ENARTHRONOTA
Eurasia_NCBI_Pterochthonius_angelus_EU432214_Atopochthoniidae_ENARTHRONOTA
Eurasia_NCBI_Gozmanyina_majesta_EU433993_Trichthoniidae_ENARTHRONOTA
Eurasia_NCBI_Atopochthonius_artiodactylus_EU432216_Atopochthoniidae_ENARTHRONOT
Outgroup_Allothyrus_sp_AF018655_Holothyrida
Outgroup_Amblyomma_boeroi_FJ464420_Ixodida
Outgroup_Opilioacarus_texanus_AF115375_Opilioacarida

Figure S1.

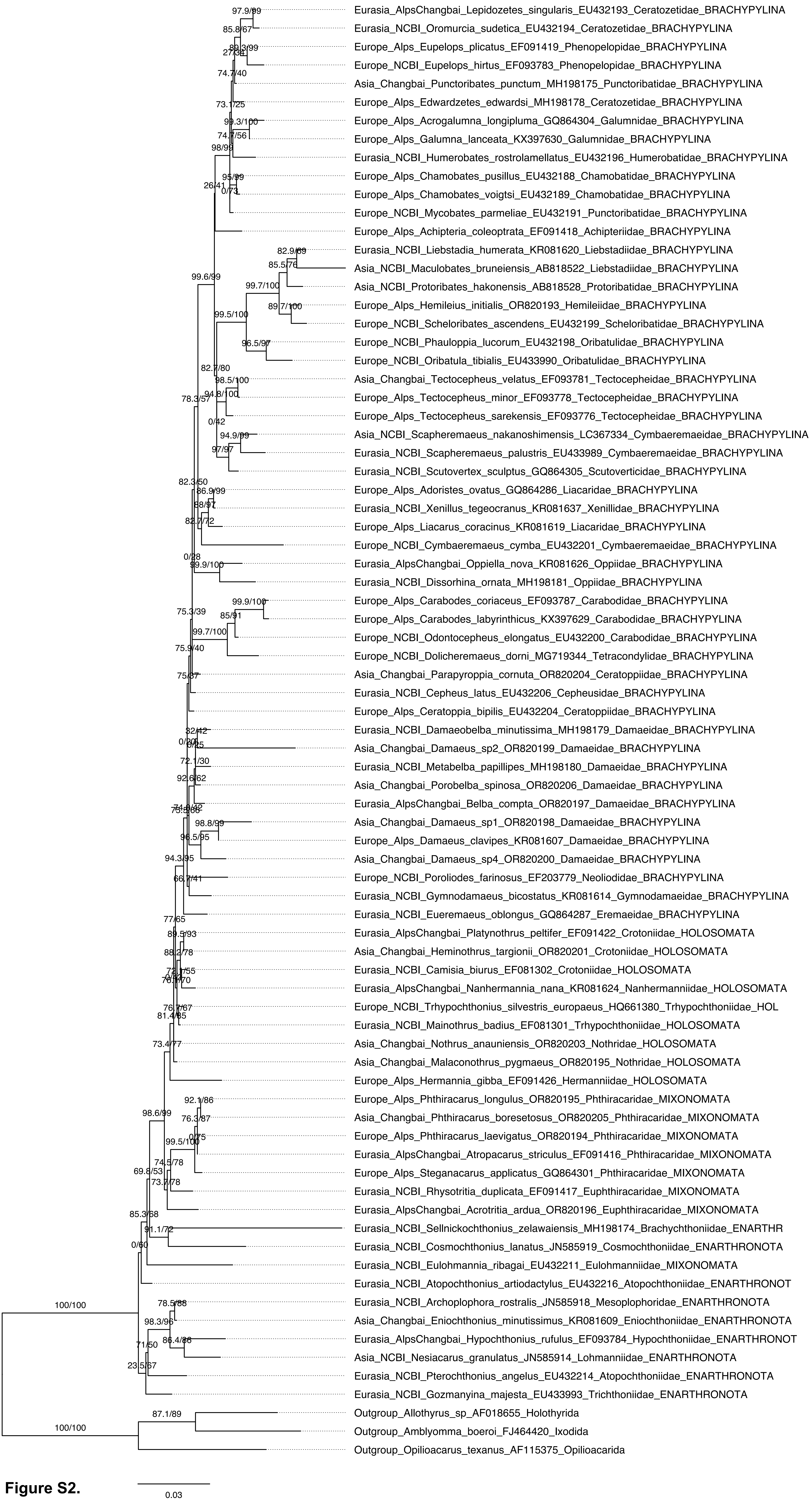
Eurasia_AlpsChangbai_Lepidozetes_singularis_EU432193_Ceratozetidae_BRACHYPYLINA
Eurasia_NCBI_Oromurcia_sudetica_EU432194_Ceratozetidae_BRACHYPYLINA
Europe_Alps_Eupelops_plicatus_EF091419_Phenopelopidae_BRACHYPYLINA
Europe_NCBI_Eupelops_hirtus_EF093783_Phenopelopidae_BRACHYPYLINA
Asia_Changbai_Punctoribates_punctum_MH198175_Punctoribatidae_BRACHYPYLINA
Europe_Alps_Edwardzetes_edwardsi_MH198178_Ceratozetidae_BRACHYPYLINA
Europe_Alps_Acrogalumna_longipluma_GQ864304_Galumnidae_BRACHYPYLINA
Europe_Alps_Galumna_lanceata_KX397630_Galumnidae_BRACHYPYLINA
Eurasia_NCBI_Humerobates_rostrolamellatus_EU432196_Humerobatidae_BRACHYPYLINA
Europe_Alps_Chamobates_pusillus_EU432188_Chamobatidae_BRACHYPYLINA
Europe_Alps_Chamobates_voigtsi_EU432189_Chamobatidae_BRACHYPYLINA
Europe_NCBI_Mycobates_parmeliae_EU432191_Punctoribatidae_BRACHYPYLINA
Europe_Alps_Achipteria_coleoptrata_EF091418_Achipteriidae_BRACHYPYLINA
Eurasia_NCBI_Liebstadia_humerata_KR081620_Liebstadiidae_BRACHYPYLINA
Asia_NCBI_Maculobates_bruneiensis_AB818522_Liebstadiidae_BRACHYPYLINA
Asia_NCBI_Protoribates_hakonensis_AB818528_Protoribatidae_BRACHYPYLINA
Europe_Alps_Hemileius_initialis_OR820193_Hemileiidae_BRACHYPYLINA
Europe_NCBI_Scheloribates_ascendens_EU432199_Scheloribatidae_BRACHYPYLINA
Europe_NCBI_Phauloppia_lucorum_EU432198_Oribatulidae_BRACHYPYLINA
Europe_NCBI_Oribatula_tibialis_EU433990_Oribatulidae_BRACHYPYLINA
Asia_Changbai_Tectocepheus_velatus_EF093781_Tectocepheidae_BRACHYPYLINA
Europe_Alps_Tectocepheus_minor_EF093778_Tectocepheidae_BRACHYPYLINA
Europe_Alps_Tectocepheus_sarekensis_EF093776_Tectocepheidae_BRACHYPYLINA
Asia_NCBI_Scapheremaeus_nakanoshimensis_LC367334_Cymbaeremaeidae_BRACHYPYLINA
Eurasia_NCBI_Scapheremaeus_palustris_EU433989_Cymbaeremaeidae_BRACHYPYLINA
Eurasia_NCBI_Scutovertex_sculptus_GQ864305_Scutoverticidae_BRACHYPYLINA
Europe_Alps_Adoristes_ovatus_GQ864286_Liacaridae_BRACHYPYLINA
Eurasia_NCBI_Xenillus_tegeocranus_KR081637_Xenillidae_BRACHYPYLINA
Europe_Alps_Liacarus_coracinus_KR081619_Liacaridae_BRACHYPYLINA
Europe_NCBI_Cymbaeremaeus_cymba_EU432201_Cymbaeremaeidae_BRACHYPYLINA
Eurasia_AlpsChangbai_Oppiella_nova_KR081626_Oppiidae_BRACHYPYLINA
Eurasia_NCBI_Dissorhina_ornata_MH198181_Oppiidae_BRACHYPYLINA
Europe_Alps_Carabodes_coriaceus_EF093787_Carabodidae_BRACHYPYLINA
Europe_Alps_Carabodes_labyrinthicus_KX397629_Carabodidae_BRACHYPYLINA
Europe_NCBI_Odontocepheus_elongatus_EU432200_Carabodidae_BRACHYPYLINA
Europe_NCBI_Dolicheremaeus_dorni_MG719344_Tetracondylidae_BRACHYPYLINA
Asia_Changbai_Parapyroppia_cornuta_OR820204_Ceratoppiidae_BRACHYPYLINA
Eurasia_NCBI_Cepheus_latus_EU432206_Cepheusidae_BRACHYPYLINA
Europe_Alps_Ceratoppia_bipilis_EU432204_Ceratoppiidae_BRACHYPYLINA
Eurasia_NCBI_Damaeobelba_minutissima_MH198179_Damaeidae_BRACHYPYLINA
Asia_Changbai_Damaeus_sp2_OR820199_Damaeidae_BRACHYPYLINA
Eurasia_NCBI_Metabelba_papillipes_MH198180_Damaeidae_BRACHYPYLINA
Asia_Changbai_Porobelba_spinosa_OR820206_Damaeidae_BRACHYPYLINA
Eurasia_AlpsChangbai_Belba_compta_OR820197_Damaeidae_BRACHYPYLINA
Asia_Changbai_Damaeus_sp1_OR820198_Damaeidae_BRACHYPYLINA
Europe_Alps_Damaeus_clavipes_KR081607_Damaeidae_BRACHYPYLINA
Asia_Changbai_Damaeus_sp4_OR820200_Damaeidae_BRACHYPYLINA
Europe_NCBI_Poroliodes_farinosus_EF203779_Neoliodidae_BRACHYPYLINA
Eurasia_NCBI_Gymnodamaeus_bicostatus_KR081614_Gymnodamaeidae_BRACHYPYLINA
Eurasia_NCBI_Eueremaeus_oblongus_GQ864287_Eremaeidae_BRACHYPYLINA
Eurasia_AlpsChangbai_Platynothrus_peltifer_EF091422_Crotoniidae_HOLOSOMATA
Asia_Changbai_Heminothrus_targionii_OR820201_Crotoniidae_HOLOSOMATA
Eurasia_NCBI_Camisia_biurus_EF081302_Crotoniidae_HOLOSOMATA
Eurasia_AlpsChangbai_Nanhermannia_nana_KR081624_Nanhermanniidae_HOLOSOMATA
Europe_NCBI_Trhypochthonius_silvestris_europaeus_HQ661380_Trhypochthoniidae_HOL
Eurasia_NCBI_Mainothrus_badius_EF081301_Trhypochthoniidae_HOLOSOMATA
Asia_Changbai_Nothrus_anauniensis_OR820203_Nothridae_HOLOSOMATA
Asia_Changbai_Malaconothrus_pygmaeus_OR820195_Nothridae_HOLOSOMATA
Europe_Alps_Hermannia_gibba_EF091426_Hermanniidae_HOLOSOMATA
Europe_Alps_Phthiracarus_longulus_OR820195_Phthiracaridae_MIXONOMATA
Asia_Changbai_Phthiracarus_boresetosus_OR820205_Phthiracaridae_MIXONOMATA
Europe_Alps_Phthiracarus_laevigatus_OR820194_Phthiracaridae_MIXONOMATA
Eurasia_AlpsChangbai_Atropacarus_striculus_EF091416_Phthiracaridae_MIXONOMATA
Europe_Alps_Steganacarus_applicatus_GQ864301_Phthiracaridae_MIXONOMATA
Eurasia_NCBI_Rhysotritia_duplicata_EF091417_Euphthiracaridae_MIXONOMATA
Eurasia_AlpsChangbai_Acrotritia_ardua_OR820196_Euphthiracaridae_MIXONOMATA
Eurasia_NCBI_Sellnickochthonius_zelawaiensis_MH198174_Brachychthoniidae_ENARTHR
Eurasia_NCBI_Cosmochthonius_lanatus_JN585919_Cosmochthoniidae_ENARTHRONOTA
Eurasia_NCBI_Eulohmannia_ribagai_EU432211_Eulohmanniidae_MIXONOMATA
Eurasia_NCBI_Atopochthonius_artiodactylus_EU432216_Atopochthoniidae_ENARTHRONOT
Eurasia_NCBI_Archoplophora_rostralis_JN585918_Mesoplophoridae_ENARTHRONOTA
Asia_Changbai_Eniochthonius_minutissimus_KR081609_Eniochthoniidae_ENARTHRONOTA
Eurasia_AlpsChangbai_Hypochthonius_rufulus_EF093784_Hypochthoniidae_ENARTHRONOT
Asia_NCBI_Nesiacarus_granulatus_JN585914_Lohmanniidae_ENARTHRONOTA
Eurasia_NCBI_Pterochthonius_angelus_EU432214_Atopochthoniidae_ENARTHRONOTA
Eurasia_NCBI_Gozmanyina_majesta_EU433993_Trichthoniidae_ENARTHRONOTA
Outgroup_Allothyrus_sp_AF018655_Holothyrida
Outgroup_Amblyomma_boeroi_FJ464420_Ixodida
Outgroup_Opilioacarus_texanus_AF115375_Opilioacarida
0.03

Figure S2.

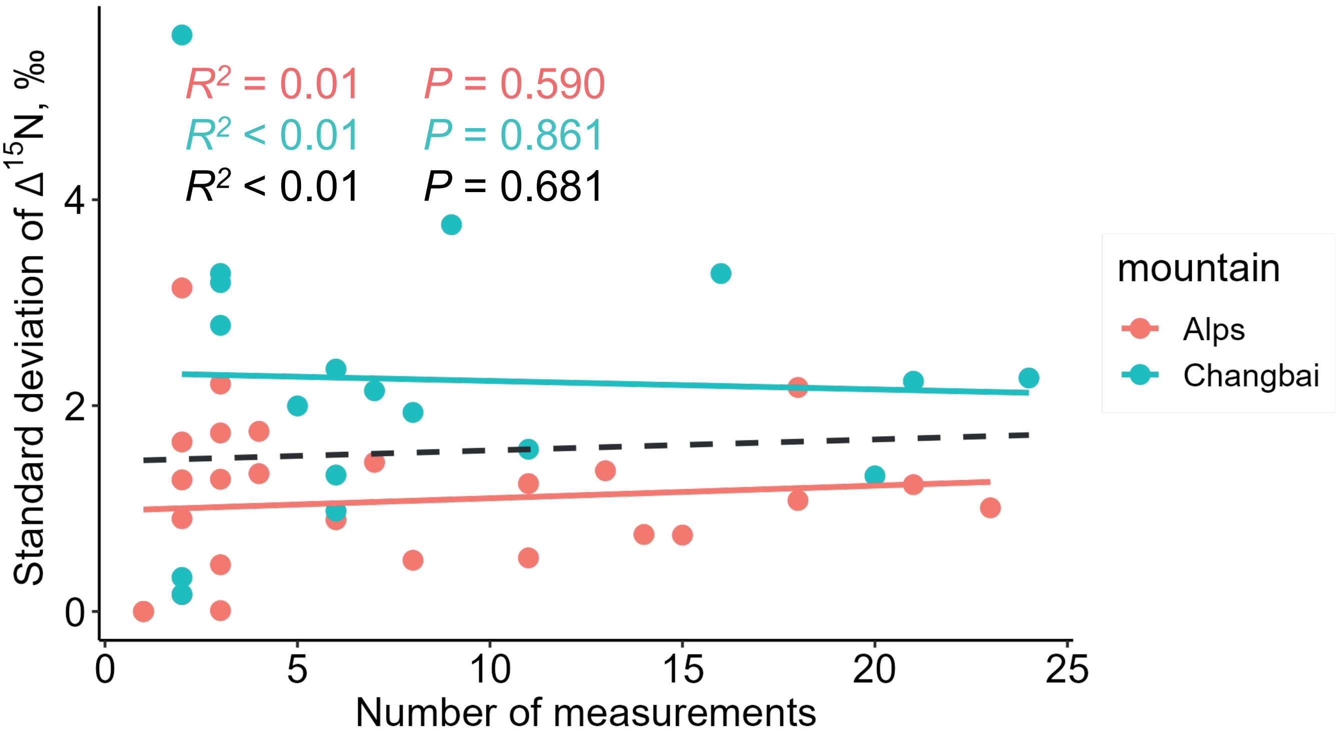

R2 = 0.01 P = 0.590
R2 < 0.01 P = 0.861
R2 < 0.01 P = 0.681
Standard deviation of Δ15N, ‰
Number of measurements
0
2
4
0
5
10
15
20
25
mountain
Alps
Changbai

Figure S3.

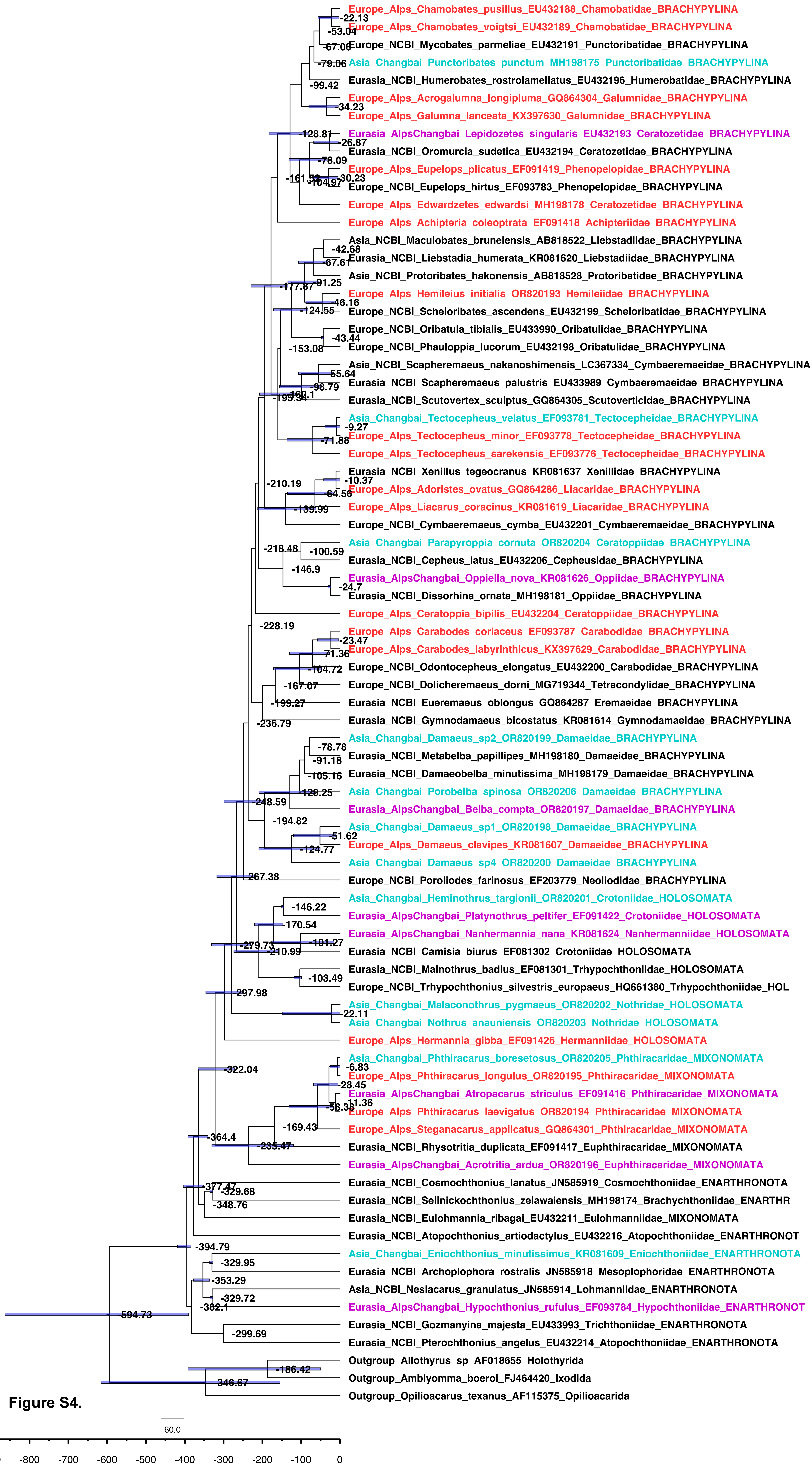
Europe_Alps_Chamobates_pusillus_EU432188_Chamobatidae_BRACHYPYLINA
Europe_Alps_Chamobates_voigtsi_EU432189_Chamobatidae_BRACHYPYLINA
Europe_NCBI_Mycobates_parmeliae_EU432191_Punctoribatidae_BRACHYPYLINA
Asia_Changbai_Punctoribates_punctum_MH198175_Punctoribatidae_BRACHYPYLINA
Eurasia_NCBI_Humerobates_rostrolamellatus_EU432196_Humerobatidae_BRACHYPYLINA
Europe_Alps_Acrogalumna_longipluma_GQ864304_Galumnidae_BRACHYPYLINA
Europe_Alps_Galumna_lanceata_KX397630_Galumnidae_BRACHYPYLINA
Eurasia_AlpsChangbai_Lepidozetes_singularis_EU432193_Ceratozetidae_BRACHYPYLINA
Eurasia_NCBI_Oromurcia_sudetica_EU432194_Ceratozetidae_BRACHYPYLINA
Europe_Alps_Eupelops_plicatus_EF091419_Phenopelopidae_BRACHYPYLINA
Europe_NCBI_Eupelops_hirtus_EF093783_Phenopelopidae_BRACHYPYLINA
Europe_Alps_Edwardzetes_edwardsi_MH198178_Ceratozetidae_BRACHYPYLINA
Europe_Alps_Achipteria_coleoptrata_EF091418_Achipteriidae_BRACHYPYLINA
Asia_NCBI_Maculobates_bruneiensis_AB818522_Liebstadiidae_BRACHYPYLINA
Eurasia_NCBI_Liebstadia_humerata_KR081620_Liebstadiidae_BRACHYPYLINA
Asia_NCBI_Protoribates_hakonensis_AB818528_Protoribatidae_BRACHYPYLINA
Europe_Alps_Hemileius_initialis_OR820193_Hemileiidae_BRACHYPYLINA
Europe_NCBI_Scheloribates_ascendens_EU432199_Scheloribatidae_BRACHYPYLINA
Europe_NCBI_Oribatula_tibialis_EU433990_Oribatulidae_BRACHYPYLINA
Europe_NCBI_Phauloppia_lucorum_EU432198_Oribatulidae_BRACHYPYLINA
Asia_NCBI_Scapheremaeus_nakanoshimensis_LC367334_Cymbaeremaeidae_BRACHYPYLINA
Eurasia_NCBI_Scapheremaeus_palustris_EU433989_Cymbaeremaeidae_BRACHYPYLINA
Eurasia_NCBI_Scutovertex_sculptus_GQ864305_Scutoverticidae_BRACHYPYLINA
Asia_Changbai_Tectocepheus_velatus_EF093781_Tectocepheidae_BRACHYPYLINA
Europe_Alps_Tectocepheus_minor_EF093778_Tectocepheidae_BRACHYPYLINA
Europe_Alps_Tectocepheus_sarekensis_EF093776_Tectocepheidae_BRACHYPYLINA
Eurasia_NCBI_Xenillus_tegeocranus_KR081637_Xenillidae_BRACHYPYLINA
Europe_Alps_Adoristes_ovatus_GQ864286_Liacaridae_BRACHYPYLINA
Europe_Alps_Liacarus_coracinus_KR081619_Liacaridae_BRACHYPYLINA
Europe_NCBI_Cymbaeremaeus_cymba_EU432201_Cymbaeremaeidae_BRACHYPYLINA
Asia_Changbai_Parapyroppia_cornuta_OR820204_Ceratoppiidae_BRACHYPYLINA
Eurasia_NCBI_Cepheus_latus_EU432206_Cepheusidae_BRACHYPYLINA
Eurasia_AlpsChangbai_Oppiella_nova_KR081626_Oppiidae_BRACHYPYLINA
Eurasia_NCBI_Dissorhina_ornata_MH198181_Oppiidae_BRACHYPYLINA
Europe_Alps_Ceratoppia_bipilis_EU432204_Ceratoppiidae_BRACHYPYLINA
Europe_Alps_Carabodes_coriaceus_EF093787_Carabodidae_BRACHYPYLINA
Europe_Alps_Carabodes_labyrinthicus_KX397629_Carabodidae_BRACHYPYLINA
Europe_NCBI_Odontocepheus_elongatus_EU432200_Carabodidae_BRACHYPYLINA
Europe_NCBI_Dolicheremaeus_dorni_MG719344_Tetracondylidae_BRACHYPYLINA
Eurasia_NCBI_Eueremaeus_oblongus_GQ864287_Eremaeidae_BRACHYPYLINA
Eurasia_NCBI_Gymnodamaeus_bicostatus_KR081614_Gymnodamaeidae_BRACHYPYLINA
Asia_Changbai_Damaeus_sp2_OR820199_Damaeidae_BRACHYPYLINA
Eurasia_NCBI_Metabelba_papillipes_MH198180_Damaeidae_BRACHYPYLINA
Eurasia_NCBI_Damaeobelba_minutissima_MH198179_Damaeidae_BRACHYPYLINA
Asia_Changbai_Porobelba_spinosa_OR820206_Damaeidae_BRACHYPYLINA
Eurasia_AlpsChangbai_Belba_compta_OR820197_Damaeidae_BRACHYPYLINA
Asia_Changbai_Damaeus_sp1_OR820198_Damaeidae_BRACHYPYLINA
Europe_Alps_Damaeus_clavipes_KR081607_Damaeidae_BRACHYPYLINA
Asia_Changbai_Damaeus_sp4_OR820200_Damaeidae_BRACHYPYLINA
Europe_NCBI_Poroliodes_farinosus_EF203779_Neoliodidae_BRACHYPYLINA
Asia_Changbai_Heminothrus_targionii_OR820201_Crotoniidae_HOLOSOMATA
Eurasia_AlpsChangbai_Platynothrus_peltifer_EF091422_Crotoniidae_HOLOSOMATA
Eurasia_AlpsChangbai_Nanhermannia_nana_KR081624_Nanhermanniidae_HOLOSOMATA
Eurasia_NCBI_Camisia_biurus_EF081302_Crotoniidae_HOLOSOMATA
Eurasia_NCBI_Mainothrus_badius_EF081301_Trhypochthoniidae_HOLOSOMATA
Europe_NCBI_Trhypochthonius_silvestris_europaeus_HQ661380_Trhypochthoniidae_HOL
Asia_Changbai_Malaconothrus_pygmaeus_OR820202_Nothridae_HOLOSOMATA
Asia_Changbai_Nothrus_anauniensis_OR820203_Nothridae_HOLOSOMATA
Europe_Alps_Hermannia_gibba_EF091426_Hermanniidae_HOLOSOMATA
Asia_Changbai_Phthiracarus_boresetosus_OR820205_Phthiracaridae_MIXONOMATA
Europe_Alps_Phthiracarus_longulus_OR820195_Phthiracaridae_MIXONOMATA
Eurasia_AlpsChangbai_Atropacarus_striculus_EF091416_Phthiracaridae_MIXONOMATA
Europe_Alps_Phthiracarus_laevigatus_OR820194_Phthiracaridae_MIXONOMATA
Europe_Alps_Steganacarus_applicatus_GQ864301_Phthiracaridae_MIXONOMATA
Eurasia_NCBI_Rhysotritia_duplicata_EF091417_Euphthiracaridae_MIXONOMATA
Eurasia_AlpsChangbai_Acrotritia_ardua_OR820196_Euphthiracaridae_MIXONOMATA
Eurasia_NCBI_Cosmochthonius_lanatus_JN585919_Cosmochthoniidae_ENARTHRONOTA
Eurasia_NCBI_Sellnickochthonius_zelawaiensis_MH198174_Brachychthoniidae_ENARTHR
Eurasia_NCBI_Eulohmannia_ribagai_EU432211_Eulohmanniidae_MIXONOMATA
Eurasia_NCBI_Atopochthonius_artiodactylus_EU432216_Atopochthoniidae_ENARTHRONOT
Asia_Changbai_Eniochthonius_minutissimus_KR081609_Eniochthoniidae_ENARTHRONOTA
Eurasia_NCBI_Archoplophora_rostralis_JN585918_Mesoplophoridae_ENARTHRONOTA
Asia_NCBI_Nesiacarus_granulatus_JN585914_Lohmanniidae_ENARTHRONOTA
Eurasia_AlpsChangbai_Hypochthonius_rufulus_EF093784_Hypochthoniidae_ENARTHRONOT
Eurasia_NCBI_Gozmanyina_majesta_EU433993_Trichthoniidae_ENARTHRONOTA
Eurasia_NCBI_Pterochthonius_angelus_EU432214_Atopochthoniidae_ENARTHRONOTA
Outgroup_Allothyrus_sp_AF018655_Holothyrida
Outgroup_Amblyomma_boeroi_FJ464420_Ixodida
Outgroup_Opilioacarus_texanus_AF115375_Opilioacarida
-22.13
-53.04
-67.06
-79.06
-99.42
-34.23
-128.81
-26.87
-78.09
-30.23
-104.97
-161.52
-42.68
-67.61
-91.25
-177.87
-46.16
-124.55
-43.44
-153.08
-55.64
-98.79
-160.1
-195.34
-9.27
-71.88
-210.19
-10.37
-64.56
-139.99
-218.48
-100.59
-146.9
-24.7
-228.19
-23.47
-71.36
-104.72
-167.07
-199.27
-236.79
-78.78
-91.18
-105.16
-129.25
-248.59
-194.82
-51.62
-124.77
-267.38
-146.22
-170.54
-101.27
-279.73
-210.99
-103.49
-297.98
-22.11
-322.04
-6.83
-28.45
-11.36
-58.39
-169.43
-235.47
-364.4
-377.47
-329.68
-348.76
-394.79
-329.95
-353.29
-329.72
-382.1
-594.73
-299.69
-186.42
-346.67
60.0
-800
-700
-600
-500
-400
-300
-200
-100
0

Figure S4.
